# Comment on “The diagnostic value of D-dimer with simplified Geneva score (SGS) pre-test in the diagnosis of pulmonary embolism (PE)”

**DOI:** 10.1186/s13019-020-01301-0

**Published:** 2020-09-10

**Authors:** Siamak Sabour

**Affiliations:** 1 Department of Clinical Epidemiology, School of Health and Safety, Shahid Beheshti University of Medical Sciences, Daneshjoo Blvd, Tehran, IR PC: 198353-5511 Iran; 2 Safety Promotions and Injury Prevention Research Centre, Shahid Beheshti University of Medical Sciences, Tehran, IR Iran

## Abstract

Any decision in clinical practice needs to evaluate both reliability (precision) and validity (accuracy) of a diagnostic test. Without knowledge about the reliability, any judgment would be wrong. In diagnostic accuracy research, it is essential to evaluate the diagnostic added value of a test, since a diagnostic accuracy of a single test might be excellent, however for clinical purposes it can be worthless. Like evaluating discrimination, it would be possible to estimate the diagnostic added value by applying ROC of diagnostic model with and without test results in the model.

To the Editor.

I read the paper from Zhihui Fu et al. published in J Cardiothorac Surg [1]. The aim of the study was to assess the diagnostic value of D-dimer with simplified Geneva score (SGS) pre-test in the diagnosis of pulmonary embolism (PE). In a retrospective analysis, 1035 patients with suspected PE were recruited. All enrolled patients were grouped according to the computed tomographic pulmonary angiogram (CTPA) results: PE patients and non-PE patients. Then, receiver operating characteristic (ROC) curve were constructed to determine the optimal D-dimer cutoff point value which is based on Yonden’s index (YI).

Although I appreciate this significant study, I would like to raise some methodological issues that can affect the interpretation of results. First, any decision in clinical practice needs to evaluate both reliability (precision) and validity (accuracy) of CTPA. Without knowledge about the reliability, any judgment would be wrong [2–6]. Second, in diagnostic accuracy research, it is essential to evaluate the diagnostic added value of CTPA, since a diagnostic accuracy of a single test might be excellent, however for clinical purposes it can be worthless. Like evaluating discrimination, it would be possible to estimate the diagnostic added value by applying ROC of diagnostic model with and without CTPA [2, 7, 8]. Hence, we suggest the authors to estimate both diagnostic added value and reliability of the CTPA by an appropriate method.

## Data Availability

NA

## Abbreviation

SGS: Simplified Geneva Score
CTPA: Computed Tomographic Pulmonary Angiogram
ROC: Receiver Operating Characteristic
YI: Yonden’s Index
